# Towards personalised molecular feedback for weight loss

**DOI:** 10.1186/s40608-019-0237-5

**Published:** 2019-05-06

**Authors:** Shilpa Tejpal, Narinder Sanghera, Vijayalaxmi Manoharan, Joan Planas-Iglesias, Kate Myler, Judith Klein-Seetharaman

**Affiliations:** 1 Systems Biology and Biomedicine, Division of Metabolic and Vascular Health, Medical School, University of Warwick, Gibbet Hill, Coventry, CV4 7AL UK; 2 Institute for Digital Healthcare, Warwick Manufacturing Group, University of Warwick, CV4 7A, Coventry, UK

**Keywords:** Obesity, Insulin, Lactate, Weight loss, Biomarkers, App development

## Abstract

**Background:**

Numerous diets, apps and websites help guide and monitor dietary behaviour with the goal of losing weight, yet dieting success is highly dependent on personal preferences and circumstances. To enable a more quantitative approach to dieting, we developed an integrated platform that allows tracking of life-style information alongside molecular biofeedback measurements (lactate and insulin).

**Methods:**

To facilitate weight loss, participants (≥18 years) omitted one main meal from the usual three-meal routine. Daily caloric intake was restricted to ~1200KCal with one optional snack ≤250KCal. A mobile health platform (personalhealth.warwick.ac.uk) was developed and used to maintain diaries of food intake, weight, urine collection and volume. A survey was conducted to understand participants’ willingness to collect samples, motivation for taking part in the study and reasons for dropout.

**Results:**

Meal skipping resulted in weight loss after a 24 h period in contrast to 3-meal control days regardless of the meal that was skipped, breakfast, lunch or dinner (*p* < 0.001). Common reasons for engagement were interest in losing weight and personal metabolic profile. Total insulin and lactate values varied significantly between healthy and obese individuals at *p* = 0.01 and 0.05 respectively.

**Conclusion:**

In a proof of concept study with a meal-skipping diet, we show that insulin and lactate values in urine correlate with weight loss, making these molecules potential candidates for quantitative feedback on food intake behaviour to people dieting.

**Electronic supplementary material:**

The online version of this article (10.1186/s40608-019-0237-5) contains supplementary material, which is available to authorized users.

## Background

Obesity is a global epidemic with increasing incidence rates in developed and developing nations. For example, the past 10 years have seen an approximate doubling in the number of obese adults in the UK alone with body-mass-index (BMI; defined as weight divided by the square of height, in kg/m^2^) values > 30. Male obesity rates rose from 13.2 to 24.4% and from 16.4 to 25.1% in women over the period 1993 to 2012 [1]. Besides body image considerations, being overweight or obese can raise blood pressure and concentrations of cholesterol, as well as cause insulin resistance. This increases the risk of developing heart disease, stroke, type 2 diabetes and certain types of cancers among other conditions [2, 3]. Thus, health-based concerns should be excellent motivating factors to lose weight. However, achieving weight loss is hard work and failure is demoralising. Most typical weight loss programs include following a diet regime (with or without drugs such as appetite suppressants), a fitness regime or a combination of these approaches. Technological support available includes diet trackers and activity monitors. Recording of eating patterns has been recognized as an effective step in managing obesity [4, 5]. While the traditional paper version of the commonly used dietary questionnaire is considered tiring [6], there are numerous computer-assisted versions [7, 8] as well as apps and websites available for personal tracking of food intake [9, 10]. However, regardless of interface, mis-reporting of food intake is a well-documented problem [6, 11], as it depends on self-awareness, honesty and motivation of the user [5]. Often, unconscious bias of self-observation leads to under-realization of foods eaten [12]. Energy expenditure on the other hand can potentially be tracked without bias using activity monitors, but they do not provide a direct link to weight loss. Even if a device that accurately measures caloric intake and expenditure was widely available, the information may not be sufficient to motivate a user to make changes in their behaviour that would result in weight loss. This is evidenced by the fact that even when meticulously keeping records of food intake, individuals still find it hard to lose weight [13]. This is because the relationship between caloric intake and weight loss is not linear [14]. As a result, current approaches to lose weight loss generally do not work well [15], and the weight loss market is missing a device that is more directly coupled to the desired outcome, weight loss. The prerequisite for such a device is a quantitative and science-based biomarker of weight loss that has the potential to provide a biological feedback loop to the dieter.

Quantitative biomarkers of weight loss do not exist yet, but biomarkers have been considered for a number of related areas. For example, urinary metabolic markers for cardiovascular disease, blood pressure and adiposity have been identified [16, 17]. Several metabolomics studies involving untargeted proton (^1^H) nuclear magnetic resonance spectroscopy and ion exchange chromatography on obese human and mice urine samples have identified metabolites associated with BMI and adiposity [16, 18]. Angiotensin converting enzyme (ACE) has been shown through blood profiling for protein and steroid hormones to be an important predictor for sustained weight loss [19]. Biomarkers for different foods include NMNA, a niacin-related (vitamin B3) metabolite marker for coffee drinking [20], proline betaine for citrus fruit consumption [21] and O-acetyl carnitine for red meat intake [22]. Thus, while biomarkers have been considered for several related purposes, none have been tested yet as a means to provide molecular feedback to someone following a diet.

The requirements for dieting feedback are more immediate than the general adiposity markers, or the long-term ACE weight loss marker. Our goal is to measure a molecular marker that directly or indirectly reports on dieting behaviour so that we can use it for feedback. We have chosen insulin and lactate because these are both known to vary with consumption of glucose and other food, as well as caloric intake overall [16, 23–25]. Caloric restriction is one of the most widely used strategies for losing weight [26, 27] but it requires strong will power, and thus identifying biomarkers of weight loss and using them for dieting feedback may make caloric restriction easier on dieters.

Besides food composition, another factor amenable to behaviour change is meal timing. Different studies have suggested that consuming a regular breakfast is associated with lower body weight [27–30]. There is also a plethora of metabolic studies that support the notion that eating breakfast is preferable over eating dinner due to a phenomenon referred to as “evening diabetes” in which insulin sensitivity is higher early during the day making it more likely to burn fat [31]. A crossover study comparing days with breakfast and lunch versus days with lunch only showed different effects on clock gene expression in healthy and type 2 diabetic subjects, while skipping of breakfast showed an altered response for clock gene profiles in both groups [32]. On the other hand several studies indicate that breakfast eaters and skippers didn’t vary significantly in terms of body weight and nutrient intake [33–36]. A study by Gill and Panda [37] has demonstrated the extent to which adults display an irregular daily and weekly rhythm of eating-fasting, which could be manoeuvred to obtain desirable health benefits. It might be possible to cater to such personal preferences, at least to some extent, without compromising weight loss success. For example, from a metabolic perspective it may be most efficient for weight loss to skip meals other than breakfast, especially dinner. However, from a behavioural and/or cultural perspective, breakfast and lunch may be the easiest meals to skip, and dinner the hardest. Weighing between these alternate decision making strategies requires quantitative analysis. Finally, to develop a tool for personalised feedback there is also a need to understand the willingness of individuals to collect samples in order to obtain metabolic biomarker information.

Here, our goal was to lay the foundation for a molecular feedback approach to assist dieting efforts. To this end, we aimed at obtaining a better understanding of the complex interplay between possible molecular biomarkers of dieting behaviour, individuals' personal preferences, their willingness to collect information, the drive to act on the collected information and weight loss success. Specifically, we asked 52 dieters to record meal timing and composition using an electronic diary interface, along with collection of urine samples, and weight. The urine samples were analysed for lactate and insulin concentrations and the information was integrated. Our results provide a strong proof of concept that each molecule studied, but especially insulin, can be used for biofeedback on dieting behaviour.

## Methods

### Study design and population

Participants were recruited by website, flyer and newsletter advertisement at the University of Warwick and through word of mouth. To be eligible for participation, individuals had to be 18 years or older and clinically healthy. Exclusion criteria included diagnosed diabetes and pregnancy. The study was approved by the Warwick Medical School Ethics committee BSREC (protocol identification REGO-2014-1318). Everyone who expressed interest in the study was referred to details on the website personalhealth.warwick.ac.uk. Those who remained interested, met with the PI of the study, and were provided with instructions on how to use the interface. We also provided them with the urine sample collection kit and instructions on how to use it and how to transfer samples to the investigators. All participants provided informed written consent at this time. Not all participants who were given the information and sample collection kit actually entered data and returned samples. Sample tubes were labelled with numbers that anonymized the samples. Data entered into the digital health platform was matched to molecular information only through the sample tube numbers.

Participants were asked to collect data for at least one 24 h period. In total, participants provided data for 149 days. The average number of 24 h periods was 3 days/participant, and the maximum number of data was entered by one participant who provided 24 periods of 24 h data each.

A control day was defined as a day in which participants provided samples and data where they consumed a regular 3-meal diet. An intervention day was defined as a day in which they provided samples and data where they skipped at least one meal. Since on average only 3 days of samples and data was provided, a washout period was not necessary.

### Demographics data collection

All participants were given the option to enter information about their age, BMI, sex, weight and ethnicity at the time of sign-up to the digital health platform. The majority of participants provided this information and this was used to carry out demographic analysis. In some cases, BMI was calculated by the investigators from weight and height entries directly. Participants were not specifically recruited to target any particular BMI. However, for analysis purposes, the data was also divided by grouping individuals according to their BMI values. Individuals with a BMI of 18.5–24.9 kg/m^2^ were considered normal; 25.0–29.9 kg/m^2^ were overweight; and those above 30.0 kg/m^2^ and greater were obese [1]. The goal of the study was not to compare obese with non-obese participants, but a separation of the data into BMI groups was useful to identify BMI as a source of variation and to delineate possible trends in the data that may relate to BMI.

### Sample collection and storage

Most samples were dropped off at the departmental front desk and the investigators collected the samples and transferred them to -80 °C upon notification by the receptionist. In some cases, samples were handed over to investigators directly by participants.

### Mobile application

The mobile and web application was developed using Intel XDK, a framework that allows building of cross-platform mobile applications, including Android, iOS and Windows along with a web browser interface (http://personalhealth.warwick.ac.uk). The programming was using standard web technologies such as HTML, Javascript and CSS. A responsive web design approach was adopted in implementing the application to enable device specific optimal user experiences. Front end browser and mobile app functionality was built using AngularJS, a Javascript framework. All back end support for the mobile app and the web interface were built using Java and MariaDB database server. Back end automation for providing users with graphical feedback was programmed using R. All data communication between the server and mobile and web applications was through HTTPs. A built-in interface between our server and the FATsecret database (https:\\platform.fatsecret.com) was developed so that study participants could seamlessly search for nutrition information without leaving our website or app. Where users did not retrieve data from the FATsecret database, investigators carried out the searches subsequently. FATsecret provides free access to their database through an API (up to 5000 calls a day were allowed). The database is very large and allows retrieval of nutritional information for the majority of food and drink items (see below).

### Meal plans

**Fig. 1:**
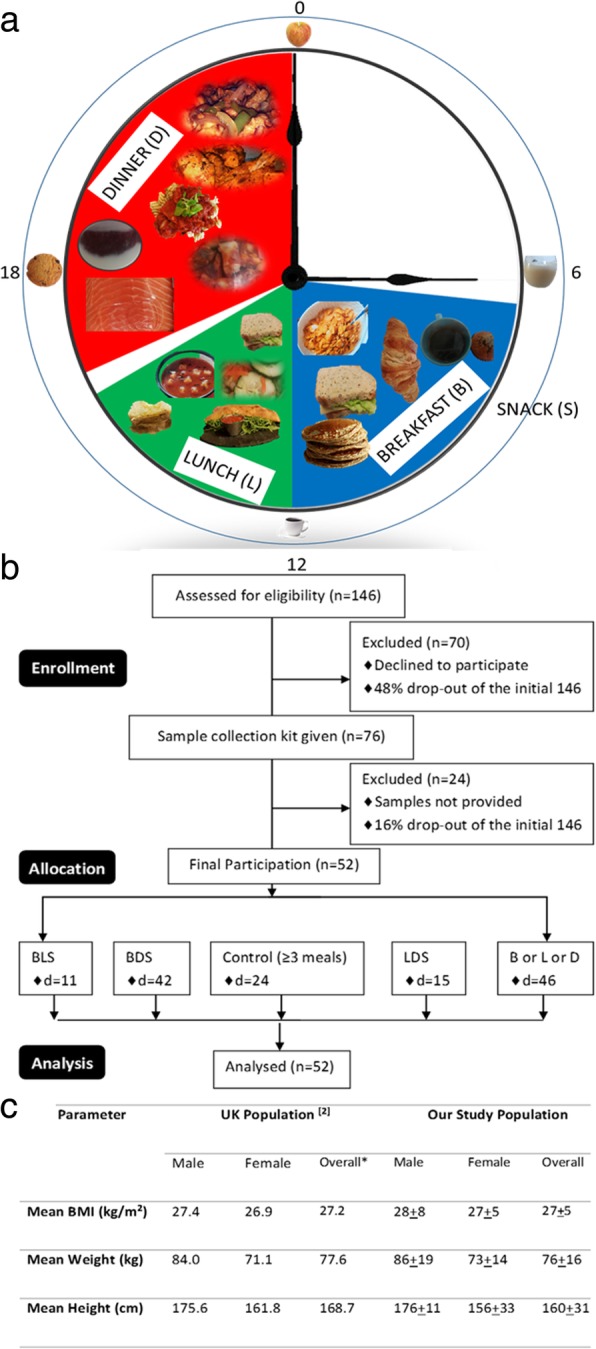
Study Design. **a** Meal plan layout for the participants in a 24 h period. **b** Flow diagram of the study design. **c** Comparison between demographic features of the study participants and the UK population. Overall numbers for the UK population was the arithmetic middle between the male and female values based on the assumption that the distribution of male and females in the statistics was approximately 50%. In this study, there was more females than males, so the overall number was obtained directly from the raw data

Participants were asked to provide data and samples for at least one control day and at least one diet day (see definition above). For diet days, participants were recommended to skip lunch (L) or dinner (D), but were also allowed to skip breakfast (B). They were also asked to restrict the total caloric intake for the day to less than approximately 1200KCal. An optional snack (S) of <250KCal was allowed once in a day. B, L, D, S were defined according to i) calorie intake (<250KCal for S, >250KCal for B, L, D) summed over nearby entries (within 30 min) and ii) timing, being B before noon, L between noon and 3 pm and D after 4 pm (Fig. 1a). A control day had three or more meals on that day. According to these specifications we defined different meal plans to systematically describe the participants’ preferences: i) B, L, [S]; ii) B, D, [S]; iii) L, D, [S]; iv) B or L or D, [S] for dieting days; and v), B,L,D, [S] or > 3 meals for control days, where [S] denotes optional snack intake (Fig. 1b).

The caloric intake was calculated  based on the meal information provided by the user. The individuals either used the pre-specified meal options from the digital health database or entered detailed descriptions of their prepared meals. The  food (in grams) breakdown in carbohydrate, fat and protein was obtained from the fatsecret database (available at https:\\platform.fatsecret.com). For analysis purposes, it was further converted into KCal by multiplying with 4, 4 and 9 per gram of carbohydrates, protein and fat, respectively. FatSecret was chosen as it had a mature API that could be used by our website and app to calculate nutrition information without maintaining our own nutrition database. In addition to offering a large and curated database of common foods and packaged products, FatSecret also offers a professional interface for health practitioners to monitor app usage of their patients [38]. It has also been previously used in several studies as a caloric counter to understand effects of tracking information (on mobile applications) on weight loss [38–40].

Entries in the database were used to determine every participants’ caloric patterns, meal plan preferences and to compare the different plans to the weight changes observed over the respective 24 h period along with insulin and lactate profiles. In all cases, only those records were analysed that contained a minimum of two meal records for the day (from 00:00 h to 12:00 h day + 1). No instructions were given to participants with respect to food composition, and any food item was allowed. Some of the food options chosen by participants are depicted in Fig. 1a.

The fasting times for diet days were obtained by subtracting the time of the second meal from the first meal of the day. Similarly, the overnight fasting period was obtained except that only those days that had following day weight information were considered.

### Insulin measurements using mesoscale luminescence assay

Insulin in samples was measured using a Mesoscale Discovery Human Insulin Kit containing (catalogue number: K151 BZC-2) 96 well plates coated with insulin antibodies obtained from Mesoscale Inc. (www.mesoscale.com). The assay was performed according to the manufacturer’s instructions. The plates were analysed on a SECTOR Imager 6000 system. All samples (urine and plasma) were centrifuged prior to analysis at 12000 rpm for 5 min at room temperature. Insulin calibrators (supplied by the kit) were run in duplicate to generate an 8-point standard curve covering the 0–50,000 pg/mL range. The standard curve was modelled using least squares fitting algorithms so that signals from standards with known concentrations of insulin can be used to calculate insulin concentrations in samples. The MSD Discovery Workbench® analysis software was used to calculate the concentration of insulin in samples. The software uses a 4-parameter logistic model and includes a 1/Y2 weighting function. This allows for a better fit of data over a wide dynamic range (3–4 logs), particularly at lower insulin concentrations. The wide dynamic range of the assay allowed for the quantification of insulin in urine without the need for dilution nor concentration.

### Lactate measurement

Lactate dehydrogenase is the enzyme responsible for interconversion of lactate to pyruvate following reduction of nicotinamide adenine dinucleotide (NAD) to its reduced form (NADH). To measure lactate, the reaction needs excess NAD. To force the reaction to completion in this direction, it is necessary to trap the formed pyruvate with hydrazine present in glycine-hydrazine buffer.

All samples were centrifuged prior to analysis at 12000 rpm for 15 min at room temperature. The experiments were conducted in 96 well solid black fluorescence plates (Thermo-Scientific, catalogue #: 634-0006). The stock solution of 10 mM of lactate was reconstituted in glycine-hydrazine buffer (0.6 M glycine and 0.5 M hydrazine, pH 9.2) bought from Sigma-Aldrich, UK. This was used to prepare standard reactions in the range of 25-100 μM lactate concentrations. A reaction mixture stock solution containing 10 mg NAD with 2.0 mL glycine buffer, 4.0 mL water 0.1 mL L-lactate dehydrogenase (Sigma-Aldrich, UK). 20 μl of standard (in duplicates) or sample and 130 μl of reaction mixture were added to each well. The plate was then incubated at 37 °C for 15 min. The fluorescence was read using a Perkin Elmer Wallac 1420 Victor2 Microplate Reader with excitation at 345-355 nm and emission at 450-460 nm.

### Survey design

A short self-administered questionnaire link was sent via email to everyone who originally expressed an interest in the study (including but not limited to those who actually enrolled), inviting them to participate in the survey. The survey was anonymous and the data from the survey was not linked or compared with the data entered by participants during the weight loss study. The questionnaire (Additional file 1: Figure S4) was designed using Google forms. A total of 48 people submitted the study questionnaire, all of whom had consented to participate in the survey and stated previous experience on entering data onto the platforms. The individuals participating in the survey were not linked to their identification in the platform, as the survey google document and digital health platform were independent of each other. The participants were provided with several options under each section (see below) along with an option to enter other factors contributing to the study.

The survey was divided into three parts:

*Section I: Motivation.* Several options were provided to the participants such as interest in losing weight, diet, metabolic profile, health platform and being involved in medical research. These parameters were analysed together and when separated into 10-year bin-sized age groups.

*Section II: Dropout.* The survey was used to identify the reasons for dropout such as difficulty of diet and sample collection, time consuming, complicated health platform. The participants were also provided a free text field to enter other factors that contributed to dropout.

*Section III: Feedback.* The participants provided feedback on the health platform and sample collection. Their personal input was requested on suggestions for the platform’s improvement.

The information provided by the individuals was used to calculate the weighted average of each contributing factor for motivation to participate, reasons for dropout and feedback about the study.

### Statistical analysis

Statistical analyses were performed using IBM SPSS Statistics 24 and R. Association between different variables was calculated using bivariate Pearson Coefficient analyses. Nonparametric Mann-Whitney U test was performed in some cases as indicated. One-way analysis of variance (ANOVA) was used to compare values from control days with values from dieting days.

## Results

### Study design and data collection

Initially, 146 individuals recruited by flyer and newsletter advertisement expressed interest in our study. Of these, 52 individuals became study participants (77% females and 23% males) who provided data and samples. They received access to a web application at personalhealth.warwick.ac.uk, as well as an app “Digital Health Platform” for android and apple devices available in google play and iTunes stores, respectively. Through this platform, they entered life-style related data, including weight, food and drink intake, exercise, and urine sample collection details. The mobile health platform creates a timeline of the logs or events that are entered by the user. This electronic information is sent to a web server that allows users to store their information securely and access it anywhere using either a web browser based interface or a native mobile application from their smart phones or tablets. In addition to being a tool for logging time and other parameters, the application also serves to seamlessly share information between the user and the analyst. It allowed researchers and cohort group participants to register, and manage the logistics of data collection. Researchers obtained analysis files in anonymized fashion only through the website administrator. Ease of use and cross-platform support were the most important among the factors considered in the design of the health platform.

Urine samples were used to measure insulin and lactate concentrations which were uploaded onto the platform. Participants collected samples and life-style data for control and diet days (see Methods).

### Demographics of study participants

According to the UK Health and Social Care Information Centre, the prevalence of overweight individuals in the UK population is age- and gender-dependent, with 9% (male) and 13% (female) in the 16–24 age group and 13% (male) and 35% (female) in the 50–69 age group [1]. A similar pattern characterized the participants in our study (Additional file 1: Figure S1). Grouping participants by age showed that the number of overweight study participants was lowest among younger adults (20–29 year old group, Additional file 1: Figure S1a), increasing through middle age (ages 30–59, Additional file 1: Figure S1b-d), and only reducing among the oldest participants (ages 60–69, Additional file 1: Figure S1e). The majority of study participants were in the normal and overweight groups (Additional file 1: Figure S1f). The mean BMI of 27.2 kg/m^2^ observed in the UK population [1] parallels that of 27.0 ± 5 kg/m^2^ (mean ± standard deviation) in our study. Similarly, the weight and height values split by gender also mirror those of the UK population (Fig. 1c). This indicates that the sample of 52 participants is a good representation of the UK population. The mean BMI of males and females in our study are 26.0 and 28.0 kg/m^2^, respectively, which indicated that they were significantly different (*p* < 0.001) within our study group (Additional file 1: Figure S2).

### Diet behaviour: caloric intake pattern

**Fig. 2:**
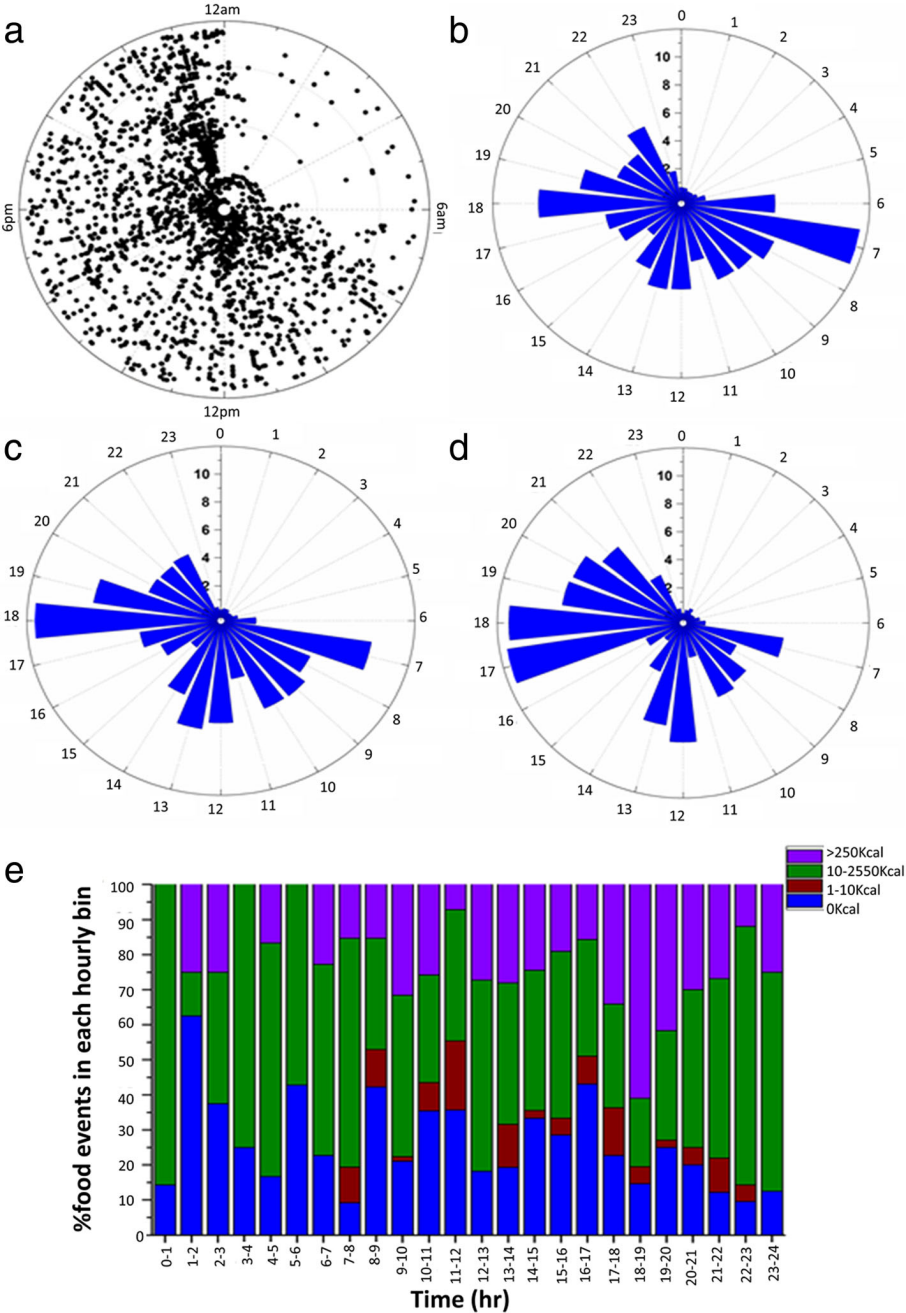
More calories are consumed at dinner and breakfast and dinner combination were more popular than breakfast and lunch. **a** Polar plot of all entries of each individual plotted against the time of day (angular axis). Data from 52 individuals are shown. 24 h rose plots showing (**b**) percentage of total entries from individuals, (**c**) percentage of ingestion events and (**d**) % of calories consumed. **e** Percentage of food events in 1 h bins. The radial axis for each rose plot shows % of events

The timings of health platform entries on the 149 days of data entered by participants show a wide spread from 7 am - midnight on a 24 h scale (Fig. 2a), with only night time (midnight to 7 am) receiving very few entries, in line with previous observations [37]. There was a higher percentage of total entries on the health platform in the mornings and evenings, namely 33 and 32% of the total entries, respectively (Fig. 2b). Many of the morning entries were weight and urine sample collections. When only food entries are plotted, entries cluster in the morning (around 7 am), at lunchtime (around 1 pm), and in the evening, peaking at 6 pm (Fig. 2c). When entries were quantified by calories consumed, one can see that the largest calorie intake was in the evening, with 22 and 51% of the total calories recorded from 7 to 11 am and 4 to 9 pm, respectively (Fig. 2d). A more detailed break-down of entries as % food events per hour is shown in Fig. 2e. Purple indicates meals, green snacks and blue/brown low calorie drinks (including water and coffee). Most entries for caloric intake of >250KCal (i.e. a meal) were observed in just one hour from 6 to 7 pm (Fig. 2e). Many breakfast (B) “meals” were low in calories and were therefore classified here as snacks (S), see below. Caloric intake is significantly different for males and females (*p* = 0.05, Additional file 1: Figure S2).

### Diet behaviour: meal plan preferences

Out of the 52 participants, at least one entry with two meals (see Methods) was available for 43 people. Since participants could freely chose the number of days they participated in the study, the number of days for which data was available varied for each participant. The majority followed the study plan for 1–2 days, while one participant collected data for up to 24 days. Thus, the 43 individuals collectively provided data for 147 days consisting of both, control (28) and diet (119) days. Participants were given a relatively free choice in meal plans, with the only restrictions being the omission of one main meal and the total caloric intake as described in Methods The meal plan choices made by participants on the 147 days is shown graphically in Fig. S3a. BL, the meal plan that would be metabolically optimal from a theoretical perspective (see Introduction), or the slightly modified BLS meal plan, were followed only on 10 days. 19 days corresponded to the LD plan, while the largest number of 46 days was in the BD or BDS category. BDS was followed on 39% of the dieting days, and was thus the most popular meal choice, while the B [S] plan accounted for only 8% of the dieting days. A graph of the spread of meal timing of individuals shows that participants followed similar eating patterns for all days if they provided samples and data for more than one day (Additional file 1: Figure S3b). Another frequently followed meal plan was that of the single meal: 46 days had only one meal B, L or D (sometimes plus optional snack, B[S] or L[S] or D[S]). This large number likely arose from the fact that we classified what participants may have thought of as “meals” as snacks based on the 250KCal cut-off. In total, there were 26 control days (18% of the 147 days), where people have had at least three meals (BLD, BLD [S] or more). On these control days, caloric intake was significantly higher (*p* < 0.01) than on diet days, as expected, although there were many days of low calorie intake among control days. Notably, individuals did not lose weight on those caloric restricted control days, suggesting that meal timing plays an important role, perhaps more than caloric intake for losing weight (Additional file 1: Figure S3c).

### Dieting success by study participants’ meal plan choice

**Fig. 3:**
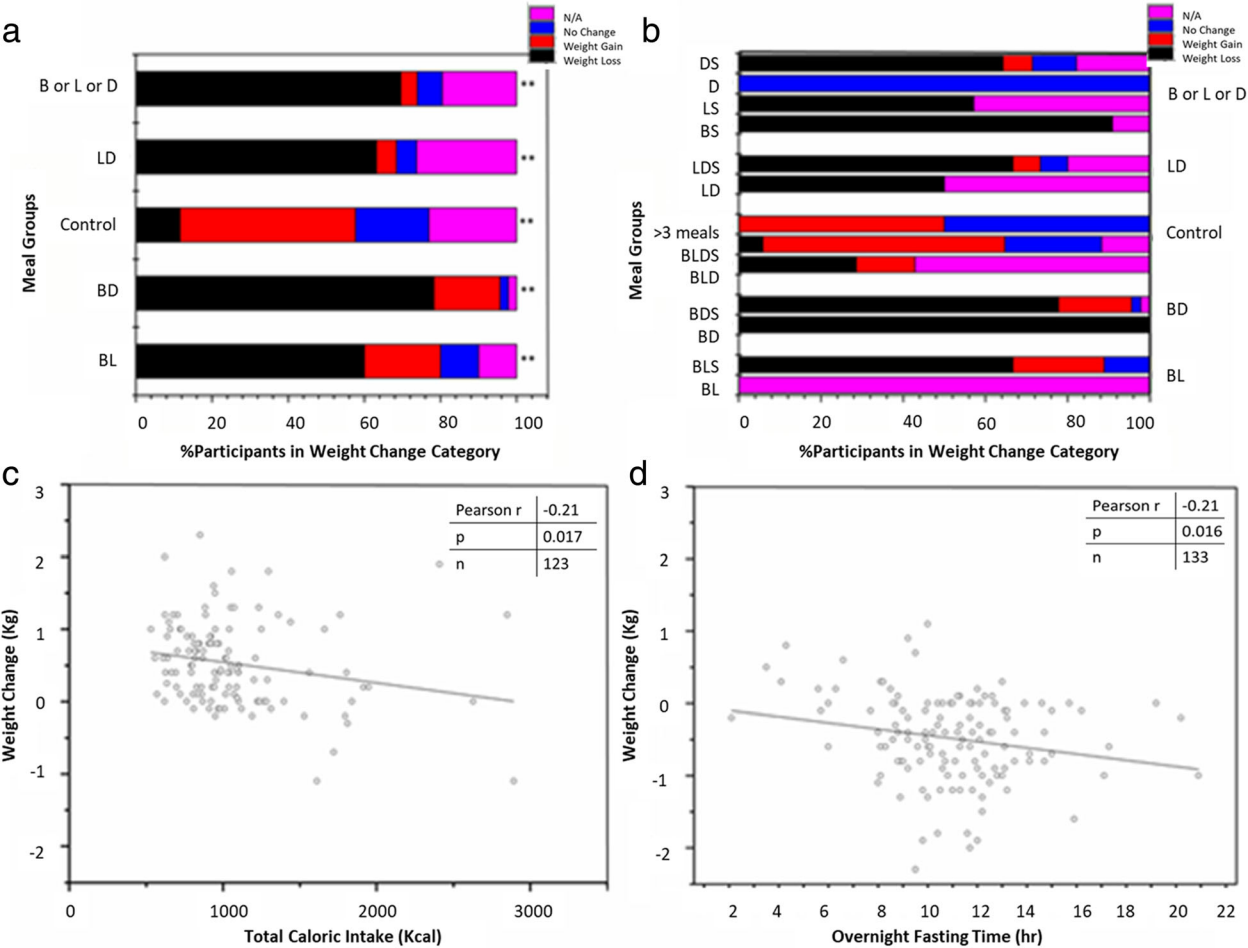
Weight loss is associated with fasting time and consumption of calories. **a** Effect of dieting on weight with respect to different meal groups. N/A refers to the days for which weight loss data is not available. Weight change is defined as weight loss (any change > 0 kg), weight gain (any change < 0 kg), and no change (=0 kg). One-way ANOVA analysis comparing control with other meal groups show significant difference at *p* = 0.01** (*p* < .001). **b** Effect of dieting on weight with respect to different meal plan subgroups. **c** Plot of total caloric intake against weight difference. Pearson’s R = − 0.21 correlation is significant at the 0.05 level (*p* < 0.05). **d** Plot of overnight fasting time against weight difference. Pearson’s R = -0.21 correlation is significant at the 0.05 level (*p* < 0.05)

Weight change data was available for only 43 out of the 52 participants for at least one 24 h period, reducing the total of 147 days to 126 days. For ease of analysis, we grouped the weight change values into 3 groups: weight loss when the weight difference between the beginning and end of the 24 h period was > 0 kg, weight gain for < 0 kg, and no change =0 kg. Figure 3a shows the % of participants with weight change in each of these groups. One can clearly see that all diet meal plans resulted more often in weight loss as compared to the control days. Figure 3b shows the more detailed split into sub-groups taking whether or not a snack was eaten into account. Overall there does not appear to be a negative consequence of having the additional snack, although the size of the data is too small to ascertain the statistical significance of this statement. Because we do have meal plan information for 21 days without weight change information, we included a fourth group “NA” (purple) in Fig. 3a, b for completeness.

We conducted one-way analysis of variance (ANOVA) comparing the control group with each of the other groups in Fig. 3a (i.e. BL, BD, LD and B or L or D). Each group was significantly different from the control group (*p* < 0.01). This indicates that skipping a meal results in weight loss irrespective of which meal of the day is skipped. Comparing weight loss with total caloric intake showed an inverse relation with Pearson Correlation significant at *p* = 0.05 (Fig. 3c). Finally, on days when participants achieved weight loss, the length of overnight fasting periods was inversely correlated to weight loss expressed as negative kg values (Pearson Correlation R = -0.21, *p* = 0.016) (Fig. 3d), i.e. the longer the fasting the greater the weight loss.

### Motivation: reasons to participate in the study

A survey was conducted to understand the reasons why people were interested in a study that involved both, weight loss and urine sample collection (Additional file 1: Figure S4). Interest in losing weight, involvement in research and knowledge of metabolic profile were the main drivers behind participation. There may be a difference in motivation for different age groups, as the 20–29 and 40–49 year age-group more often reported interest in their metabolic profile (33%), while the 30–39, 50–59 and 60–69 year age groups were more motivated by losing weight (32, 32 and 20% respectively). However, because of the small number of participants, we cannot ascertain if these differences are statistically significant.

### Dropout analysis

Dropout rates in weight loss studies have been a prominent concern when promoting lifestyle and dietary changes in overweight and obese populations, as well as affecting the validity and generalisation of conclusions in weight loss studies [41]. In our study, we observed a similar trend. At the first meeting, people were informed of the study requirements. At this stage, of the 146 adults who had shown an interest, 70 dropped out, leaving only 76 individuals who provided written consent for participation in the study and received sample collection kits. Of these, 52 participants actually provided urine samples and life-style information through our online/mobile platforms. Thus, the dropout rate after the first meeting of 48% reduced to 16% when comparing to the initial number of people interested in the study, and 34% when comparing to the previous step (Fig. 1b).

To identify the reasons for dropout in our study, we designed a number of questions (Additional file 1: Figure S4). Busy schedule, complicated samples collection and loss of motivation correspond to 25, 21 and 18% of the reasons chosen by people who participated in the survey, respectively. Apart from pre-defined reasons, individuals also entered their personal hurdles through a free text option. Participants found it hard to follow caloric restriction guidelines due to their active work life or the psychological stress given by the word “diet”. The fear of eating more after a day of dieting also made people drop out from the study. In addition, since the individuals in our study were UK based, they found it difficult to maintain the food diaries as the fatsecret database we used was an American foods database.

### Molecular insulin and lactate biomarker correlate with life-style data

**Fig. 4:**
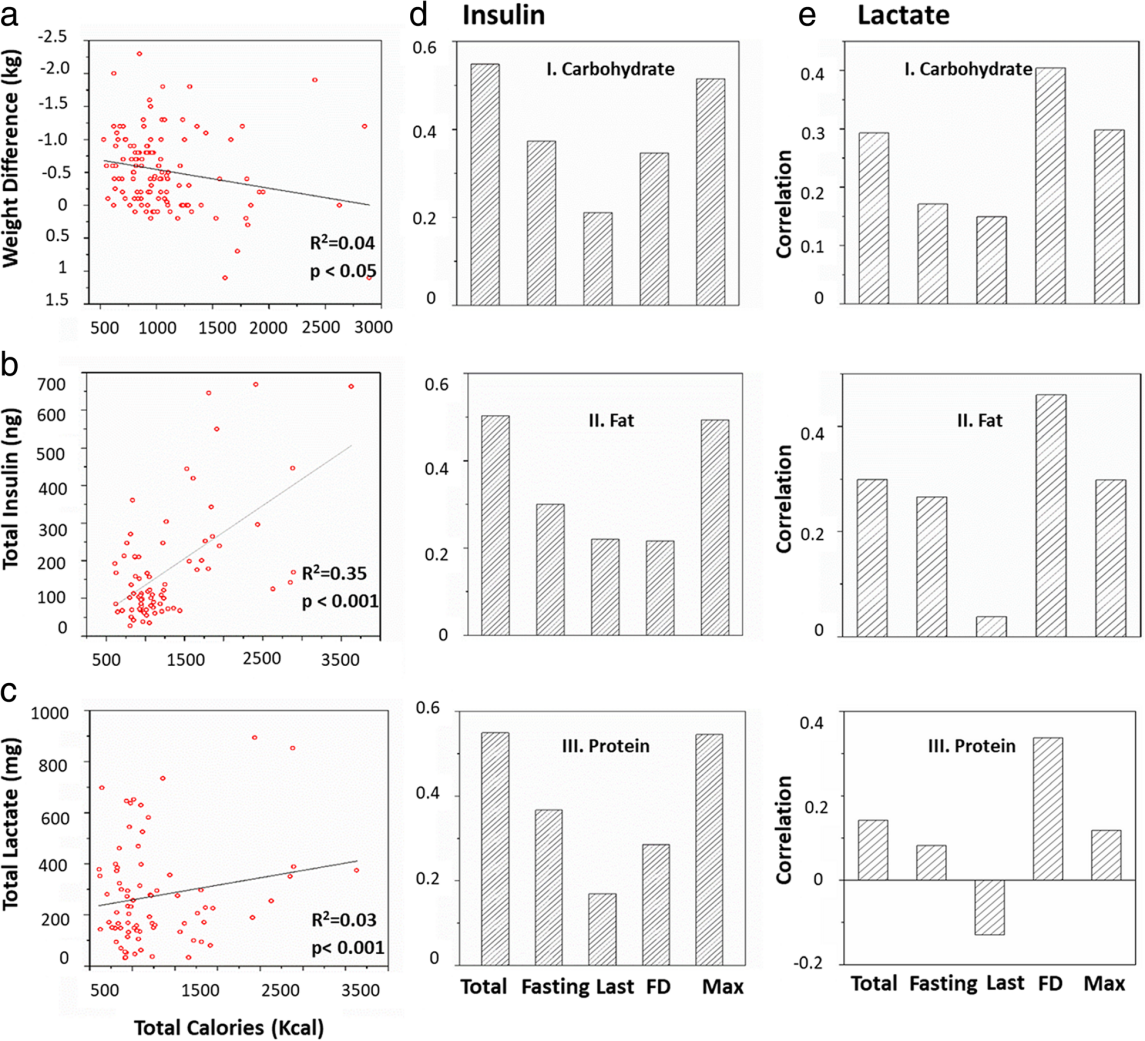
Individual correlation plots of selected parameters. **a** Weight difference versus total calories. **b** Total insulin versus total calories. **c** Total lactate versus total calories. **d** Insulin parameters correlation with nutritional parameters: Panel I. Carbohydrate. Panel II. Fat. Panel III. Protein. **e** Lactate parameters correlation with nutritional parameters, as in (**d**). Significant correlations with macronutrient content were marked by ** or *, when significant at *p* = 0.01** and *p* = 0.05*, respectively

**Fig. 5:**
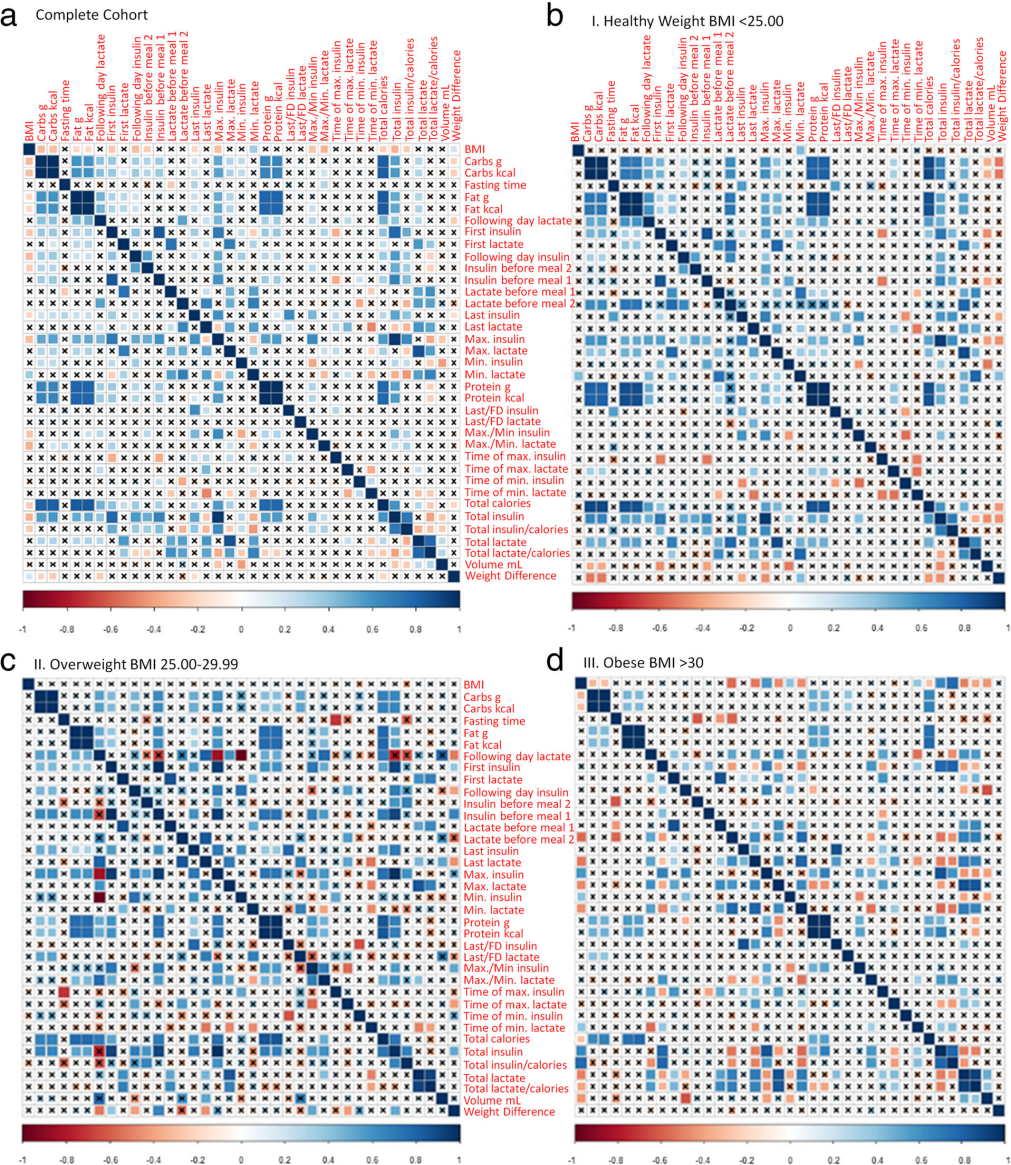
Correlation plot of measured variables. **a** The correlation (or lack thereof) between the parameters is shown for 147 days. Correlations between the parameters were scaled from 1.0 to − 1.0. Blue indicates positive correlation while red indicates negative correlation. X indicates no correlation between the two parameters. **b** Correlation plot of measured variables for heathy individuals with BMI up to 25 (panel I), in the overweight category with BMI in the range 25–30 (panel II) and the obese category with BMI > 30 (panel III)

The urine samples collected by the participants were used to measure insulin and lactate concentrations. These values were then used to extract a total of 23 parameters relating to biomarker profiles or lifestyle data entered (Additional file 1: Figure S5). The cross-correlation matrix of all the 23 extracted parameters from biomarker profiles and the digital health platform are shown for the complete cohort in Fig. 5a for an overall summary. The weight difference showed a positive correlation with BMI while a negative correlation with carbohydrates, fat, lactate before second meal of the day and total calories was observed. Furthermore, as expected, the total lactate and insulin parameters were strongly correlated with other parameters such as first, last, maximum, minimum and following day lactate and insulin concentrations. In particular, the weight difference (expressed as negative kg) showed a correlation with total calorie intake, which was significant at R = 0.04 (*p* < 0.05). Total insulin and total lactate were positively correlated to the total calorie intake (*p* < 0.001, R = 0.35 and R = 0.03, respectively). Fasting, total, last, following day and maximum amounts for insulin and lactate had significant correlation with carbohydrate, fat and protein content in the meals (Fig. 4d, e).

### Biomarker and BMI

**Fig. 6:**
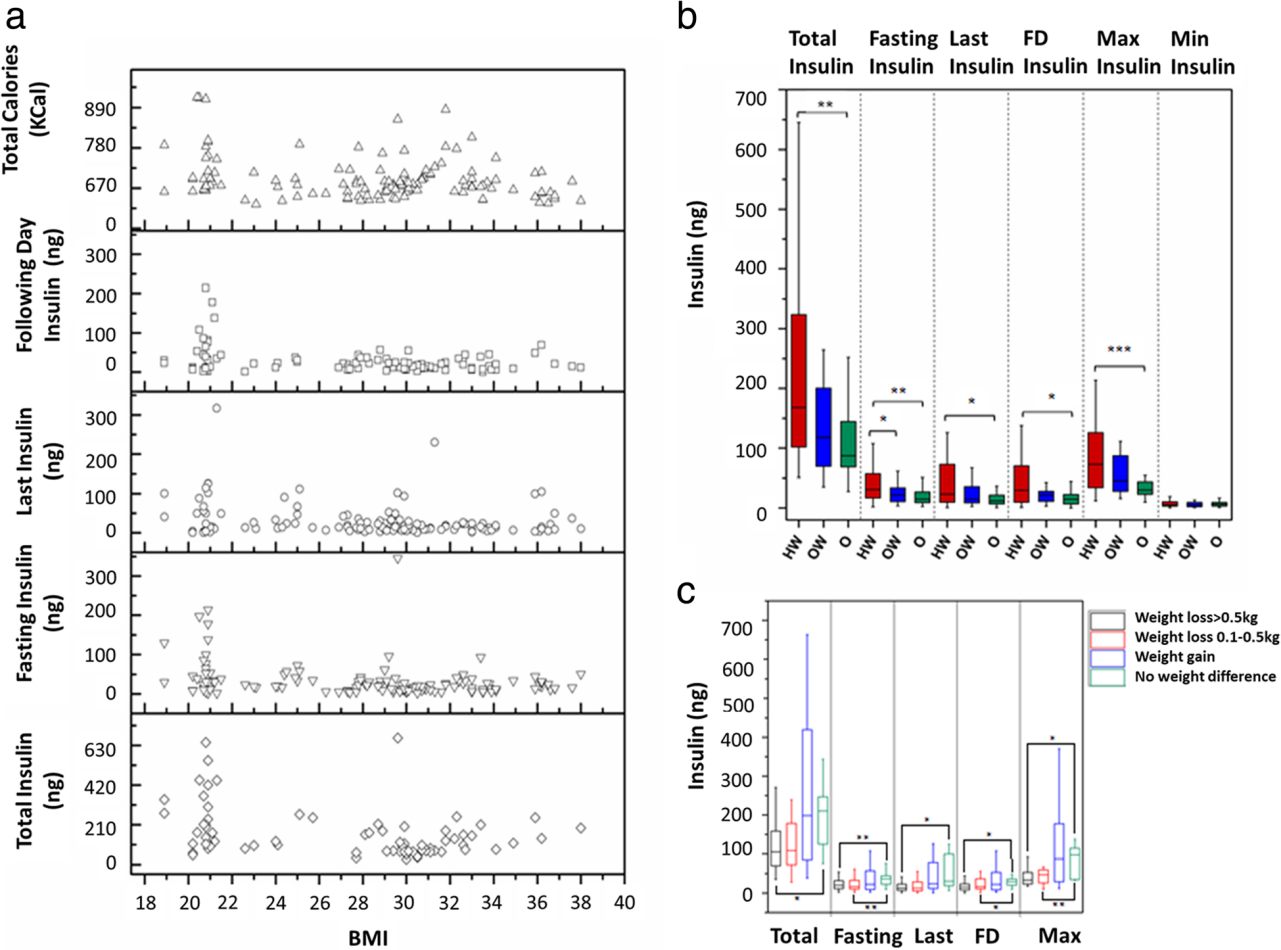
Insulin response is dependent on BMI. **a** Spread of total, fasting, last, following day insulin and total calories of all the participants in comparison to BMI. **b** Comparison of insulin parameters among healthy, overweight and obese participants. Significance levels are marked as follows: *p = 0.05, **p = 0.01, p = < 0.001. **c** Weight loss is associated with low insulin values. Weight change was grouped into four groups, no weight difference, weight gain or weigh loss between 0.1–0.5 kg and > 0.5 kg. Significance levels are marked as follows: *p < 0.05; **p < 0.01

Because BMI was correlated with a number of parameters (Fig. 5), we investigated if pre-defined BMI groups differed in correlation of parameters (Fig. 5b). Segregation of the data into different BMI groups showed loss of correlation between weight loss and other parameters in the obese and overweight groups while being sustained in the healthy group. Particularly, the insulin biomarker profiles in the overweight and obese group are dampened in comparison to the healthy group (Fig. 6a). Total, fasting, last, following day and maximum insulin values were significantly higher in the obese group in comparison to healthy individuals (Fig. 6b). Also, total and last lactate amounts increased in obese people in comparison to the healthy group (Additional file 1: Figure S7a). Furthermore, total, maximum and minimum lactate values were higher in obese than in overweight individuals (Additional file 1: Figure S7b), in accordance with previous findings of increased lactate levels in obese individuals [42].

### Biomarker and weight loss

Because weight loss is the desired outcome for most dieters, we plotted weight change versus biomarkers in Fig. 6c. Because of inaccuracies inherent to measuring weight, we grouped the weight change values into 4 groups: weight loss > 0 .5kg, weight loss 0.1–0 .5kg, weight gain and no change. One can see very clearly that total insulin values vary most dramatically in the weight gain group, and are overall higher in the no weight and weight gain categories. Similar patterns were also observed for fasting, last, following day and maximum insulin values. This graph thus emphasizes that insulin values, even individual ones, as opposed to all values collected over a 24 h period, are potentially useful biomarkers for immediate feedback on dieting behaviour, with low values being likely predictive of weight loss, information which can only be obtained the day following a diet, too slow to be sufficiently motivating.

## Discussion

The escalating obesity epidemic that may in part even be related to the recent decline in life expectancy in the USA [43, 44] requires novel approaches suitable to help people lose weight. In this paper, we describe the first attempt at developing quantitative, molecular feedback mechanisms for people dieting. While biofeedback is well established to be successful in diabetes [45], it has not been studied in people with no obvious signs of a disease. Our approach also differs from previous efforts at identifying biomarkers of sustained weight loss which had for example identified ACE levels, amongst others [19]. While extremely useful, this information is long-term, and cannot be used for immediate feedback to dieters. The present paper fills this gap. For the first time, we demonstrate, that metabolic markers can be used in conjunction with food intake behaviour and have the potential to predict weight loss. Thus, a person on a diet, in the future, can measure their insulin (or to a lesser extent, lactate) values and make a decision if it is acceptable to eat another meal that day, or what type of meal it should be. Our current study has provided the proof of concept that biomarker measurements can be used in this context. Limitations of our study are the short term nature of the diet (24 h periods, as opposed to more realistic weeks/months of dieting) and the length and cost of the assay of insulin, and the need for urine samples. Thus, both assays for urine require a laboratory setting, making it not yet feasible to conduct a long-term study or investigate the effect on behaviour. We are currently in the process of developing a rapid, cheap and home-based sensor for insulin and lactate [46], which will enable us to address these limitations in the future. As the majority of participants only provided data for 2–3 times 24 h periods, a long term trial is needed to demonstrate if similar conclusions can be reached over longer periods of dieting.

Our study was intended as a proof of concept to demonstrate if molecular measurements may provide useful information during dieting efforts. The most useful information for a dieter is weight loss. Thus, the main purpose of the study was to identify if there may be any correlation between molecular data and weight loss. Because this was an observational study with a relatively small number of participants (52), the treatments (which meal to skip and on what day) were not assigned randomly. Thus, the protocol of measurement, as well as sampling may cause the study not to be representative of the general population. Sources of sampling bias could be due to this being a volunteer sample, as well as a convenience sample imposed by the requirement to transfer urine samples to the laboratory for measurements. The bias associated with this was made evident by the large disparity between male and female participants (77% female, 23% male). Consequently, there may be a sampling bias because participants were not chosen at random and they might exhibit different lifestyles. Since most participants worked or studied at the University of Warwick, participants cannot be considered representatives of the UK population (although some of the demographics were similar), nor could the conclusions necessarily be extrapolated to people from other countries. Another source of sampling bias introduced by the observational nature of the study is that participants were given the liberty to choose what days to diet, as well as what meals to skip. This has resulted in different meal plans to be followed for different number of days by the individuals. Therefore, there is another instance of non-probability sampling, thus creating a possibility for statistical bias. There are also sources of response bias because participants were asked to record their data in an app, this means that participants may forget or neglect to record data. Also, participants could have entered incorrect values for meal calories, thus indicating voluntary response bias. Another form of response bias, more unique to this study, was improper measurements bias by the participants. Users were asked to record their weight, as well as collect samples of their urine. Incorrect sample storage, and errors in measuring urine volume, improperly weighing themselves, or using a poor scale could all result in inaccurate entries in the digital health platform. Individuals entered information on the health platform for 147 days but weight information was only provided for 126 days. The missing weight information for those 20 days could have affected the dieting success and biomarker levels and meal plan choice. Incorrect use of weighing machine, height measurement by the participants could have resulted in misclassification of individuals in BMI groups. Finally, no conclusions on causation was intended or can be inferred due to the high likelihood of confounding variables. One such variable is the fact that some individuals recorded data on consecutive days, while others on single days separated by days without data entries. There could have been an effect on some of the measurements after consecutive days of skipping meals. This, in turn, might have affected the conclusions of the study. Another possibly confounded variable was which meal was omitted. For example, participants may have skipped a meal where they regularly ate a lot as opposed to skipping a meal where they regularly eat less. Also, other daily activities might also have had an effect on an individual’s weight loss and thus been confounded with other variables in the study. While the platform contained an entry form for physical activity, few entries were made. In summary, the present study contained a number of sources for potential bias that can be addressed in future efforts. Most importantly, the data collected provides us with the necessary information to design a larger study in which we can randomly assign participants to meal plans over a longer period of time. Given that the last insulin and lactate measurements of the day are the most informative, a future study can restrict sample collection to these samples, allowing for data collection over an entire diet period which normally takes place over several weeks. Once these molecular measurements can be carried out by participants directly at home, recruiting participants not only from the university campus would allow broadening of the participant profiles.

Extensions to the study can also include improvements to our digital health platform. Our current app provides the setting that allows recording of life-style related data, including weight, food and drink intake, exercise, and urine sample collection details. It also provides automation for the analysis of the data. To broaden the use of the app additional access to local based food information databases need to be included (such as TESCO/Sainsbury’s basket for UK users). Increase in user-friendliness of the app could also help to target a wider audience. With the wide-spread use of smartphones and tablets, apps that run on these devices have become a structural part of our lives [47]. 74% of European and 73% of American adolescents use a smartphone on a regular basis [47]. With the increase in abundance of such technologies came the development of fitness and health apps that can provide behavioural interventions [47, 48]. However, Alley et al. (2017), have shown that there are only 25 apps that directly target sedentary behaviour, physical activity and/or diet. No app so far provides personalised feedback using molecular measurement information. This is the gap the approach described in this paper is aiming to fill, which we hope could help target behaviour change techniques in individuals, or in obesity clinics, weightwatcher programs and other organizations that aim to assist individuals or patients making life-style changes.

## Conclusions

In this study, we have investigated a molecular feedback approach to assist dieting efforts and behavioural responses of people using a web- and mobile-based application to assist weight loss efforts. We found that skipping a meal in a day regardless of which one, while also recording all food and exercise events that day and collecting urine samples for subsequent molecular profiling, resulted in consistent weight loss for that day, in comparison to control days in which any number of meals was allowed. Insulin and lactate values show correlations to BMI, caloric patterns and weight differences. In particular, low insulin and lactate values are likely predictive of weight loss. This could be sufficiently motivating to dieters, a hypothesis that needs to be tested in a future behavioural study and over longer periods of dieting.

## Additional files

Additional file 1: **Supplementary Information.** The file includes figures supporting the results of the paper. (DOCX 1132 kb)

Additional file 2: **Raw Data.** The file includes the raw data collected during the study. (XLSX 46 kb)

## Abbreviations

ACE: Angiotensin converting enzyme
B: Breakfast
BMI: Body-mass-index
D: Dinner
L: Lunch
NAD: Nicotinamide adenine dinucleotide
S: Snack
