# Supplementary material for: Towards personalised molecular feedback for weight loss
Source: BMC Obes. 2019 May 6;6:20. doi: 10.1186/s40608-019-0237-5 (PMC6501287; doi:10.1186/s40608-019-0237-5)

**Towards Personalised Molecular Feedback for Weight Loss**

Shilpa Tejpal^1^, Narinder Sanghera^1^, Vijayalaxmi Manoharan^1^, Joan Planas-Iglesias^1^, Kate Myler^1^ and Judith Klein-Seetharaman^1,2*^

^1^Division of Metabolic and Vascular Health, Medical School, University of Warwick, Gibbet Hill, Coventry, CV4 7AL, UK

^2^Institute for Digital Healthcare, Warwick Manufacturing Group, University of Warwick, Coventry, CV4 7A, UK

**Supplementary Figure S1: Demographic of study participants.** Comparison of BMI of participants across different age groups (20-29, a; 30-39, b; 40-49, c; 50-59, d; 60-9, e). Comparison of BMI of participants across all age groups. BMI values are available for 31 participants out of 52. Significance levels are marked as follows: *p<0.05; **p<0.01.


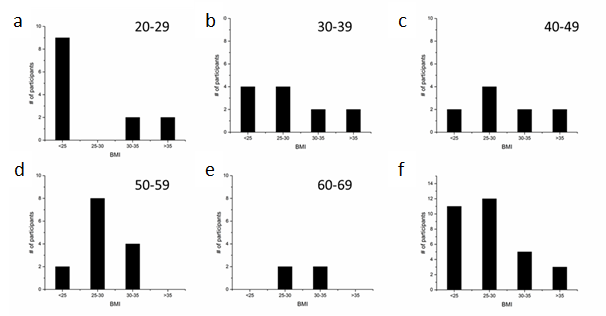


**Supplementary Figure S2:** **Gender Differences.** Comparison of different parameters across male and female groups with significance at p=0.05.


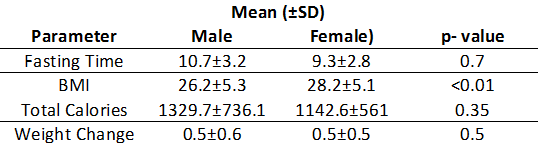


**Supplementary Figure S3: Skipping a meal resulted in weight loss.** (a) Distribution of meal plans. The meal plan preference is calculated by assigning each day to a meal plan category (BL (“Plan 1”), BD (“Plan 2”), LD, Control and B or L or D) based on the definitions described in Methods. (b) Eating duration of individuals, error bars are standard deviations where individuals have provided more than one day data. (c) Total caloric consumption by 52 participants across meal groups (BL, BD, Control, LD and B or L or D). **Caloric intake is significantly different between group at p=0.01.


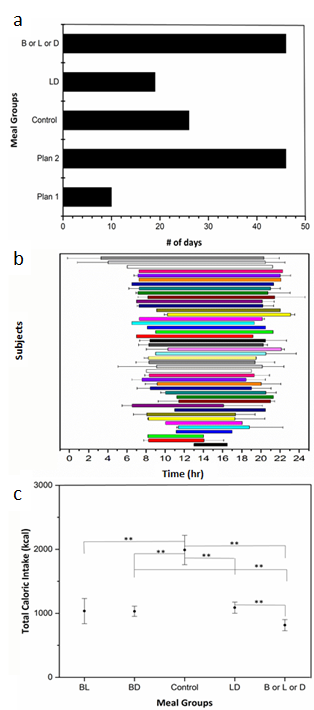


**Supplementary Figure S4: Survey Questionnaire.** Questionnaire designed with input from the research team and generated on google forms. For i), ii), ix) and xv) participants were allowed to choose more than one option.


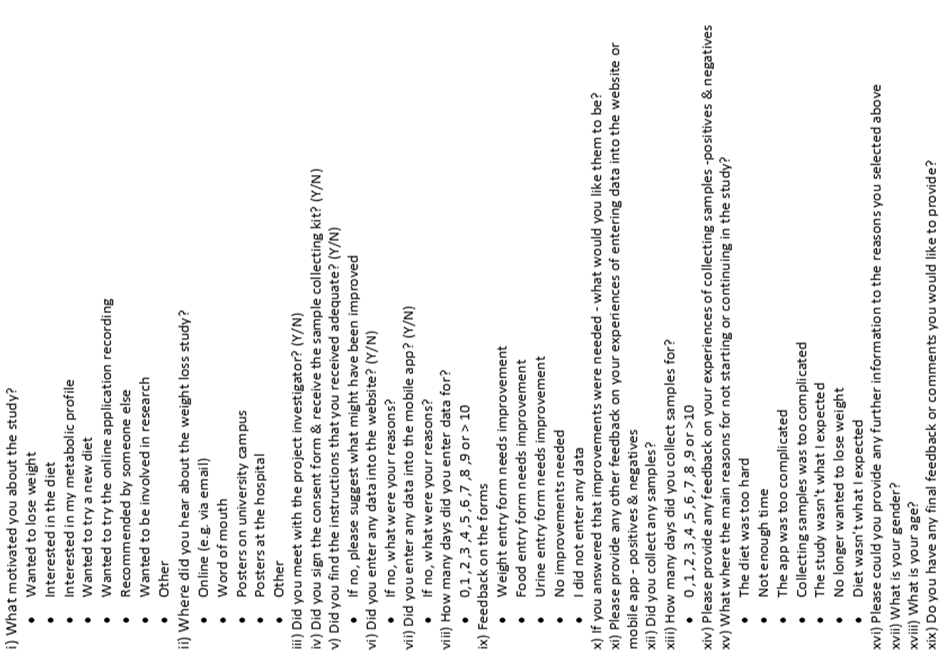
**Supplementary Figure S5:** Variables extracted from biomarker profiles and entries on the digital health platform (<https://personalhealth.warwick.ac.uk/>).

**Supplementary Figure S6:** Correlation plot of measured variables for data from one individual who provided samples and data entries for 24 days.


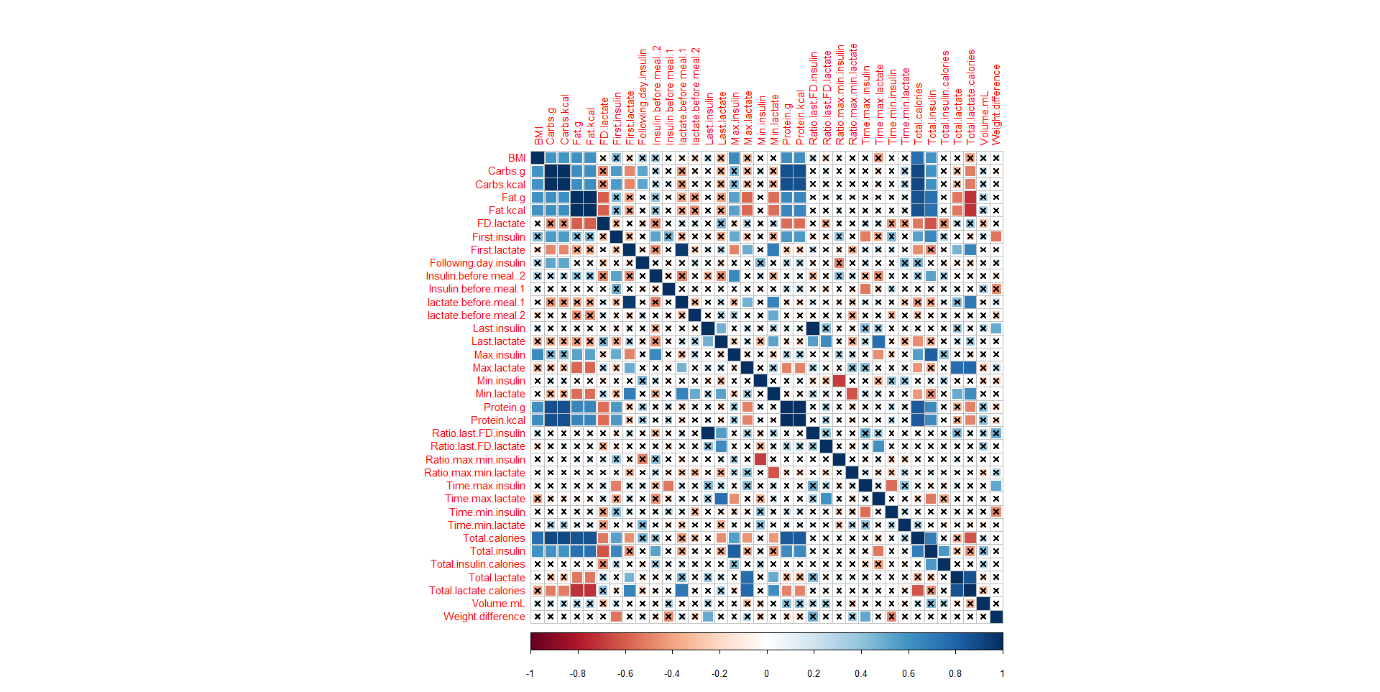


**Supplementary Figure S7: Lactate response is dependent on BMI**. (a) Spread of total, fasting, last, following day lactate and total calories of all the participants in comparison to BMI. (b) Comparison of lactate parameters among healthy, overweight and obese participants. Significance levels are marked as follows: *p=0.05.**p=0.01.p=<0.001.


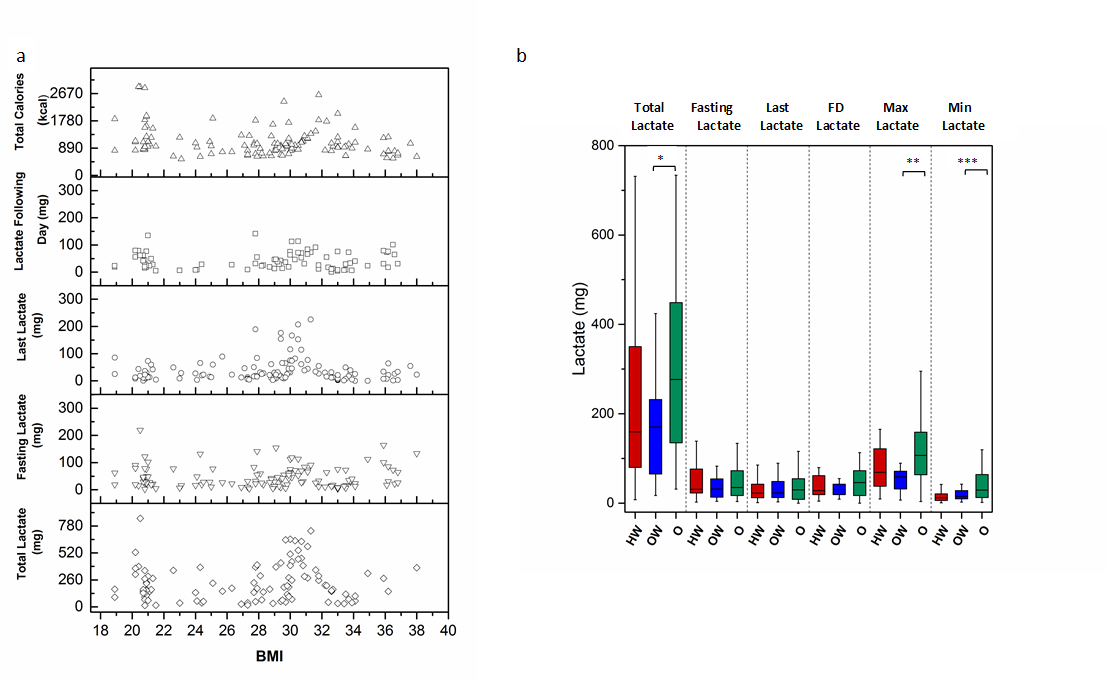

Supplement: Supplementary file 1 — Supplementary Information. The file includes figures supporting the results of the paper. (DOCX 1132 kb) [file 40608_2019_237_MOESM1_ESM.docx]
